# Do we always need to treat patients with spinal muscular atrophy? A personal view and experience

**DOI:** 10.1186/s13023-020-01593-4

**Published:** 2021-02-11

**Authors:** Caterina Agosto, Eleonora Salamon, Antuan Divisic, Francesca Benedetti, Luca Giacomelli, Aashni Shah, Giorgio Perilongo, Franca Benini

**Affiliations:** 1 Paediatric Palliative Care, Pain Service, Department of Women’s and Children’s Health, University of Padova, Padua, Italy; 2 Pediatric Residency Program, University of Padova, Padua, Italy; 3 Polistudium srl, Milan, Italy; 4 Paediatric Neurology, Department of Women’s and Children’s Health, University of Padova, Padua, Italy

**Keywords:** Spinal muscular atrophy, Ethics, Palliative care, Treatment, Nusinersen

## Abstract

**Background:**

We report the clinical outcomes observed in our patients with SMA type 1 or 2 receiving nusinersen, and we comment on the ethical implications of this treatment, in line with our results and those reported by Audic et al. in their analysis published in the Orphanet Journal of Rare Diseases.

**Methods:**

We analyzed records of all children with a genetically diagnosed SMA and clinically confirmed diagnosis of SMA Type 1 or 2 to whom nusinersen was offered. Follow-up lasted 30 months.

**Results:**

Among the 17 children with SMA type 1, 6 interrupted treatment with nusinersen due to adverse events or lack of efficacy. Of the remaining 11 patients, 9 are responding to therapy, though multidisciplinary complex care is still required. All those children started nusinersen at a very early age.

Eighteen patients with SMA type 2 received nusinersen; five required treatment interruption. The other 13 patients are still on nusinersen therapy, and 6 are responders. Among the seven non-responders, only two met the inclusion criteria of the pivotal trial.

**Conclusions:**

Our analysis further supports the findings reported in the study by Audic et al. We believe that a wider use of nusinersen in clinical practice would require a comprehensive assessment of its actual benefits weighed against the discomfort caused to patients, as well as the identification of the patients who may obtain the best benefits from this treatment.

## Introduction

Nusinersen is an antisense oligonucleotide able to enhance the synthesis of a functional SMN protein in the central nervous system [1]. This molecule has shown to prolong survival after 2 years of age in different populations of infants with spinal muscular atrophy (SMA) [2–5]. However, nusinersen requires an intrathecal administration, and the benefits potentially associated with this molecule can be somehow offset by increased use of invasive treatments [6–8].

Indeed, as recently suggested by Audic et al. in their excellent study recently published in *Orphanet Journal of Rare Diseases*, nusinersen can change the natural history and the standard care of children affected with SMA, especially in those with severe forms of disease, and in younger ones. However, patients treated with nusinersen remain disabled and continue to require intensive care [6]. Furthermore, the anxiety and distress caused by the life-long administration of a burdensome procedure, associated with the frustration due to the lack of perceived improvements, can have a major impact on the child and family’s quality of life. Last, the cost of the drug and of the hospital admissions should be considered [9, 10].

Here, we report the clinical outcomes observed in our patients with SMA type 1 or 2 receiving nusinersen, and we comment on the ethical implications of this treatment, following our results and those reported by Audic et al. in their analysis.

## Methods

### Setting

We report here the descriptive analysis of chart review (February 2018 to June 2020) of records of all children with a genetically diagnosed SMA and clinically confirmed diagnosis of SMA Type 1 or 2 [11, 12] to whom nusinersen was offered at our Institution (University Hospital of Padua, Padua, Italy). Patients were either included within the Expanded Access Program (EAP) of nusinersen or received the drug after its approval (October 2017). The Local Ethical Committee has approved this analysis and guardians of all patients have signed an informed consent to the use of child’s data for research purposes.

### Standard practice

Due to the multidisciplinary expertise required to manage children with SMA and the life-threatening nature of the disease, at our Institution all children with SMA are included since diagnosis within a palliative pediatric care (PPC) program and are followed by the local PPC Service, which coordinates the entire PPC of the Veneto region (northern Italy). Different professionals with experience in PPC were involved in the management of patients with SMA: Pediatricians, Nurses with competence in pediatric intensive care medicine, one specialist in Rehabilitation medicine and one Physical Therapist.

Once the diagnosis of SMA is established by a genetic test, our standard practice is to set a meeting between the multidisciplinary team (Pediatric Neurologist, Genetics Specialist, Pediatric Palliative Care Specialist, Psychologist) and the parents of the patient, in order to discuss the clinical status of the child and the possible strategies to be implemented. During the meeting, the clinicians provide the family with information on the disease and its natural history and describe the available therapeutic options and the complexity of care. Real-life experiences of other children and their families are also described. Usually parents request few days to think about the condition of their child and their future projects; in a second meeting, the multidisciplinary team and the parents decide together to select a proactive approach or rather a palliative one. Clinical data and child’s and parent’s perception of the course of treatment are collected at every evaluation, and the treatment plan can be reviewed accordingly at any time. The assistance of the local Ethical Board is sought as needed.

### Administration of nusinersen

Nusinersen is administered according to the following schedule: loading dose (12 mg/5 mL) within one week from communication of diagnosis, then the same dose at day 14, 28 and 63; then, maintenance doses (12 mg/5 mL) every 4 months (120 days). The patients are fully sedated before the administration of the drug and rachicentesis is performed by a trained neurologist or anesthesiologist with experience in pediatrics. Before the administration of the loading dose and then at every visit, patients were evaluated by the CHOP scale [4] if diagnosed with SMA type 1 or HMFSE scale [5] or the HINE scale [13], as appropriate.

Home visits are regularly performed, and patients are followed in terms of postural rehabilitation, nutritional care, respiratory support, pulmonary acute care, and end-of-life management as needed.

## Results

### SMA type 1 population

In total, 19 children with SMA type 1 were offered nusinersen; two families (10%) refused to start the therapy and decided to follow the natural history of the disease with the only palliative care approach.

Among the 17 children who started the nusinersen infusions, 11 (65%) were included in the EAP (3 males) and returned to our Center to continue the treatment. Of the 17 patients with SMA type 1, 9 (53%) were diagnosed with SMA type 1C, 6 with SMA type 1B (35%) and 2 (12%) with SMA type 1A, according to the international classification proposed by Finkel et al. [11]. Median age at first dose (L1) was 10 months (range 2 months–16 years) and disease duration at first dose was 4 months (range 2 months–15 years).

Among the 11 children included in EAP, two met the clinical criteria for inclusion in the pivotal trials (age 9 months and 13 months, diagnosis of SMA 1B and 1C, respectively), while the remaining nine did not (eight required ventilatory support, of whom two continuous invasive ventilation; for the remaining patient, family was not deemed as adequate).

The 17 patients with SMA1 were observed for a period of 30 months. During this period, three families out of the 17 involved decided to interrupt treatment with nusinersen, in two cases after an episode of acute respiratory insufficiency requiring invasive care and home ventilatory support; in the remaining case, the patient herself, a 12-year-old girl, asked for treatment interruption together with her family due to adverse events (refractory hypokalemia). In three other cases, patients were not responding to therapy according to the CHOP scale [4] and were offered the opportunity to interrupt treatment. In two of these cases, the children’s families agreed with the interruption of therapy and palliative care was instituted. In the remaining case, the family moved the child to another center for the prosecution of therapy, but the patient died at home two weeks after the last dose of nusinersen.

The other 11 patients remain on nusinersen therapy. Of them, 9 (81% of those still on treatment) are responding to therapy and show reduced requirement of ventilation, though multidisciplinary complex care is still required. All those children started nusinersen at a very early age, when they were pauci-symptomatic. The other two patients are not responding to therapy but are still receiving nusinersen while waiting for the approval of less invasive treatments.

### SMA type 2 population

Eighteen patients with SMA type 2 received nusinersen; median age at first dose (L1) was 10 years (range 1–15 years) and disease duration at first dose was 9 years (range 1–14 years).

Over the 30 months of observation, five (28%) patients, who were all layers in terms of function, required treatment interruption. This decision was taken, after extensive discussion with the patient and his/her family, in one case for child’s own expression of will, in the other cases by shared decision of the patients and his/her caregivers. All patients were not responders according to the HMFSE or the HINE scales [5, 13].

The other 13 patients are still on nusinersen therapy. Of them, 9 were sitters in terms of functionality (69% of those still on treatment), and 6 (46%) were responders (1 layer; only one patients still required nocturnal non-invasive ventilation). Among the 9 sitters, 5 were responders, with age ranging from 8 months to 5 years; the child aged 5 years showed high functionality at diagnosis and was able to take a few steps. Of the four sitters who were not responders, only one remained stable in terms of functionality according to the HMFSE scale; the remaining three showed a progressive decline in functionality.

Among the seven non-responders, with an age range of 5–13 years, only two met the inclusion criteria of the pivotal trial (2–12 years of age) [5]. The younger, aged 5 years, was not able to stand, but only to sit, at diagnosis.

No patient experienced adverse events due to the drug, and one patient required prolonged hospitalization due to a procedural adverse event.

## Discussion

In the PPC setting, we continuously facing incurable illness with limited treatment opportunities. The very same definition of ‘incurability’ can be challenging, due both to the unpredictable trajectory of the underlying illness and to the introduction of new therapies into the market [14].

In this respect, spinal muscular atrophy can be considered a paradigm: the recent approval of nusinersen can offer patients an enhanced duration and quality of life [2–6]. However, its actual benefits should be weighed against the need for invasive treatments, continuous and complex care, and finally costs.

In their landmark study, conducted in France on a large (N = 204) population of patients with SMA type 1 or 2, Audic et al. [6] showed that most patients remained severely disabled, and none achieved walking ability. Remarkably, in that study, the improvements in motor function were greater in patients who were children < 6 years of age, thus suggesting that early initiation of treatment can be associated with more evident efficacy on functionality.

Our analysis, although conducted in a less formal fashion and on a much lower number of patients compared with the study by Audic et al., further supports the findings reported in the French study. Indeed, a large proportion of our patients with SMA type 1 interrupted therapy, in one case according to patient’s own expression of will, and approximately 20% of those continuing therapy were not responders. However, although without any statistical analysis to fully support this statement, patients who initiated treatment early gained some more benefits compared with those on a delayed initiation.

In addition, with respect to patients with SMA type 2, a large proportion of patients required treatment interruption. Overall, the patients who were meeting the criteria for eligibility to treatment of the pivotal trial [5] showed more favorable outcomes.

## Conclusions

In the PPC setting and especially for children with rare diseases, we have the ethical duty to deeply evaluate the appropriateness of any medical interventions, with the aim to respect the best interests and the quality of life of the child [15, 16]. We believe that a wider use of nusinersen in clinical practice would require a comprehensive assessment of its actual benefits weighed against the discomfort caused to patients, as well as the identification of the patients who may obtain the best benefits from this treatment. Furthermore, the burden for caregivers should be evaluated by means of dedicated questionnaires.

## Data Availability

Not applicable.

## Abbreviations

SMA: Spinal muscular atrophy
EAP: Expanded Access Program
PPC: Palliative pediatric care
